# Efficient Coagulation Removal of Fluoride Using Lanthanum Salts: Distribution and Chemical Behavior of Fluorine

**DOI:** 10.3389/fchem.2022.859969

**Published:** 2022-03-04

**Authors:** Xiaocong Zhong, Chen Chen, Kang Yan, Shuiping Zhong, Ruixiang Wang, Zhifeng Xu

**Affiliations:** ^1^ Faculty of Materials Metallurgy and Chemistry, Jiangxi University of Science and Technology, Ganzhou, China; ^2^ State Key Laboratory of Separation and Comprehensive Utilization of Rare Metals, Guangzhou, China; ^3^ Jiangxi College of Applied Technology, Ganzhou, China

**Keywords:** fluoride removal, LaF_x_^3-x^ complexes, coagulation, colloidal particles, precipitation

## Abstract

**Abstract:** La-loaded absorbents have been widely reported for fluoride removal due to the strong affinity of La^3+^ towards fluoride ion. Herein, chemical removal of fluoride from flue gas scrubbing wastewater using lanthanum salt is investigated. The retaining free F^−^ concentration, phase composition and morphology of filtration residues, and the distribution of fluorine have been investigated using ion-selective electrode, analytical balance, scanning electron microscopy, and X-ray diffractor. The results show that at La/F molar ratio ≥1:3.05, the majority of fluorine exists as LaF_*x*_^3−*x*^ complexes, leading to the failure of fluoride removal. At 1:3.20 ≤ La/F molar ratio ≤1:3.10, the formation of LaF_3_ is facilitated. However, co-existing LaF_*x*_^3−*x*^ tends to absorb on the surface of LaF_3_ particles, leading to the formation of colloidal solution with large numbers of LaF_3_·LaF_*x*_^3−*x*^ suspended solids. At an optimized La/F molar ratio of 1:3.10, a fluoride removal of 97.86% is obtained with retaining fluorine concentration of 6.42 mg L^−1^. Considering the existing of positively charged LaF_*x*_^3−*x*^ and LaF_3_·LaF_*x*_^3−*x*^, coagulation removal of fluoride is proposed and investigated using lanthanum salts and negatively charged SiO_2_·*n*H_2_O colloidal particles, which is *in-situ* provided *via* Na_2_SiO_3_ hydrolysis at pH near 5.5. At a La/F molar ratio of 1:3.00 and Na_2_SiO_3_ dose of 0.50 g L^−1^, a fluoride removal of 99.25% is obtained with retaining fluorine concentration of 2.24 mg L^−1^. When Na_2_SiO_3_ dose increases to 1.00 g L^−1^, the retaining fluorine concentration could be further reduced to 0.80 mg L^−1^.

## Introduction

Fluorine is one of the main contaminants in ground water and industrial effluents (Kong et al., 2020; Wan et al., 2021). Long-term intake of high-fluoride level water may lead to dental fluorosis, bone fluorosis and even neurological damage (Díaz-Flores et al., 2021). According to a standard issued by the World Health Organization (WHO), the fluorine content in drinking water should be less than 1.5 mg L^−1^ (Kim et al., 2020).

Following the large-scale exploitation of sphalerite ores in recent decades, the available sphalerite ores present a decreasing grade and contain complicated components (Hu et al., 2017). Among the typical impurities in sphalerite ores, the fluoride has attracted extensive attention due to its detrimental effects on the peel-off of cathodic product (O'Keefe and Han, 1992) and the corrosion of lead-based anodes during the zinc electrowinning process (Zhong et al., 2015; Zhong et al., 2017). During the roasting of sphalerite ores, about 70% of the fluorine and chlorine in the ores enter the flue gas, and the others incorporate into the zinc calcine. In order to further reduce the content of fluorine and chlorine, the zinc calcine is usually treated in a rotary/tube furnace at elevated temperature, where fluorine and chlorine would be volatilized into flue gas in the form of HF and HCl, respectively (ÇinarŞahin et al., 2000; Kahvecioglu et al., 2013). Similarly, the zinc recovery processes from Waelz oxide, zinc oxide fumes, electric arc furnace dust, and other zinc-bearing dusts also discharge flue gas containing HF and HCl (Wei et al., 2010; Martins et al., 2021). During the scrubbing of above-mentioned flue gas, fluorine and chlorine transfer into the liquid phase, yielding a large amount of fluorine-containing acidic scrubbing wastewater. Due to the potential threat of fluorine on human’s health, the fluoride ions must be selectively removed before being discharged. According to China’s ‘Emission Standard of Pollutants for Copper, Nickel and Cobalt Industries’ (GB25467-2010), the fluoride ion content in discharge water should be below 5.0 mg L^−1^ (Liu., 2021).

Varied literatures have reported methods to efficiently remove fluoride from aqueous solutions, such as chemical precipitation (Turner et al., 2005; Chang and Liu., 2007; Huang et al., 2017), coagulation (Sandoval et al., 2019), adsorption (Ravuru et al., 2021; Yang et al., 2020; Sadhu et al., 2021), ion exchange (Samadi et al., 2014) and membrane separation (Feng et al., 2008; Arahmana et al., 2016). Among these methods, chemical precipitation has been widely used in metallurgical industry due to its advantages of simple operation, low cost, and applicability for high-fluorine wastewater. At present, the most widely used precipitant for fluoride removal is lime. However, due to the limitation of solubility product of CaF_2_, it is difficult to achieve a retaining fluoride content below 7.5 mg L^−1^ (Wajima et al., 2009) via lime precipitation, which is higher than the regulation limit of fluoride level.

As a highly electropositive metal, La^3+^ has a strong affinity towards fluoride ion (Nagaraj et al., 2017). Consequently, a large number of La-loaded absorbents have been reported for fluoride removal from ground water (G.J. Millar et al., 2017; Yan et al., 2017; Wang et al., 2018; He et al., 2019). Absorption has been regarded as one of the most effective methods to treat ground water with relative low fluoride concentration (<100 mg L^−1^), which features low retaining fluoride concentration, low cost, and easy operation (Zhou et al., 2018). In a typical flue gas scrubbing wastewater, the fluoride content could be higher than 300 mg L^−1^, La-loaded absorbents are not suitable to treat this aqueous solution because of its relatively small absorption capacity, which could lead to a large consumption of absorbents and long operation time. Hence, chemical precipitation of fluoride ion using lanthanum salt was proposed to remove fluoride in this work. In spite of extensive report on La-loaded absorbents, the distribution and chemical behavior of fluorine in the presence of La^3+^ remains unclear.

In the present work, the aqueous equilibrium diagrams of F-H_2_O and La-F-Cl-H_2_O systems were made to understand the species distribution of fluorine and lanthanum element in La-F-Cl-H_2_O system at varied pH values. The retaining fluorine concentration, precipitate morphology and structure, and distribution of fluorine in La-F-Cl-H_2_O system at different La/F molar ratios (1:3.20 ≤ La/F molar ratio ≤1:2.40) were investigated and analyzed. Based on the experimental results mentioned above, the distribution and chemical behavior of fluorine in the presence of La^3+^ was discussed in detail. Furthermore, coagulation strategy was proposed to efficiently remove fluoride by adding Na_2_SiO_3_ and La(NO_3_)_3_·H_2_O. The retaining fluorine in the equilibrium solution can be reduced to 0.80 mg L^−1^.

## Experimental Section

### Reagents

NaF, NaCl, La(NO_3_)_3_·6H_2_O, Na_2_SiO_3_, NaOH, HNO_3_ of analytical grade were purchased (Sinopharm Group, China) and used without further purification. The fluorine-containing synthetic solutions were prepared to stimulate flue gas scrubbing wastewater with NaF, NaCl, and deionized water, and its pH was adjusted to 2.0 ± 0.1 using HNO_3_. The fluorine and chlorine concentrations of synthetic solutions were set as 300 mg L^−1^ and 1,200 mg L^−1^ respectively, closing to those of the industrial flue gas scrubbing wastewater.

### Aqueous Equilibrium Diagram

The aqueous equilibrium diagrams of F-H_2_O and La-F-Cl-H_2_O systems were made using Hydra database and MEDUSA^©^ software. Specifically, the Hydra database (Puigdomenech, 2004) provided thermodynamic data such as composition data, possible solid phases formed, potential chemical reactions and their equilibrium constants. In the F-H_2_O system, the main species considered were HF, F, H_2_F_2_, HF_2_^−^, H^+^ and OH^−^. In the La-F-Cl-H_2_O system, F-, HF, H_2_F_2_, HF_2_^−^, La^3+^, LaF^2+^, LaF_2_^+^, LaF_3_, LaF_4_^−^, La(OH)^2+^, La(OH)_2_^+^, La(OH)_3_, La(OH)_4_^-^, La_5_(OH)_9_^6+^, LaCl^2+^, LaCl_2_^+^, H^+^ and OH^−^ were taken into account. The chemical reactions and corresponding equilibrium constants are listed in Table 1. These data were sent to MEDUSA^©^ software to make diagrams based on principles of mass conservation, simultaneous chemical equilibrium, and electronic charge neutrality. The distribution of fluorine species in the F-H_2_O system, and the distribution of La or F species in the La-F-Cl-H_2_O system were analyzed.

**TABLE 1:** Chemical reactions in La-F-Cl-H_2_O aqueous system and their equilibrium constants.

Equation no	Reaction	Lg K
(1)	2H^+^ + 2F^−^ = H_2_F_2_	6.77
(2)	H^+^ + F^−^ = HF	3.18
(3)	H^+^ + 2F^−^ = HF_2_^−^	3.62
(4)	La^3+^ = H^+^ + La(OH)^2+^	−8.66
(5)	La^3+^ = 2H^+^ + La(OH)_2_^+^	−18.14
(6)	La^3+^ = 3H^+^ + La(OH)_3_	−27.91
(7)	La^3+^ = 4H^+^ + La(OH)_4_^-^	−40.86
(8)	5La^3+^ = 9H^+^ + La_5_(OH)_9_^6+^	−71.20
(9)	La^3+^ + F^−^ = LaF^2+^	3.85
(10)	La^3+^ + 2F^−^ = LaF_2_^+^	6.65
(11)	La^3+^ + 3F^−^ = LaF_3_	8.69
(12)	La^3+^ + 4F^−^ = LaF_4_^−^	10.35
(13)	La^3+^ + Cl^−^ = LaCl^2+^	0.29
(14)	La^3+^ + 2Cl^-^ = LaCl_2_^+^	−0.03
(15)	H_2_O = H^+^ + OH^−^	−14.00

### Experimental Procedure and Characterization

The pH of the fluorine-containing synthetic solution was firstly adjusted to 5.5 ± 0.1 using NaOH. Afterwards, different amounts of La(NO_3_)_3_·6H_2_O were added to 200 ml of synthetic solution under agitation (200 rpm) based on a specific La/F molar ratio. The fluoride removal reaction proceeded for 1 h at 30 ± 1°C. During the reaction, fluoride ion selective electrode and pH meter were used to monitor the variation of pF (-log(a_F-_), measured continuously without pH adjustment) and pH value with reaction time. At the end of reaction, the solution was vacuum filtrated using inorganic filtration membrane (pore diameter of 0.22 μm, Jinteng®). The filtration time was recorded, and the filtration residue was collected. The final pF of the solution was measured using ion selective electrode after adjusting the pH of filtrate to 5.5 ± 0.1. The filtration residues collected were dried overnight in an oven at 80°C. After this, the weights of filtration residues were obtained using weight loss method. The morphologies and phase structures of filtration residues were investigated using scanning electron microscopy (SEM, MIRA 3) and X-ray diffraction (XRD, MiniFlex 600-C). At a La/F molar ratio ≥1:3.05, there was no precipitate obtained, extra NaF (0.133 g) was added into the filtrate to produce LaF_3_ precipitates, which were further collected, dried, weighed and analyzed as mentioned above.

Removal of fluoride from synthetic solution using Na_2_SiO_3_ and La^3+^ was also investigated. Firstly, different doses of Na_2_SiO_3_ (0.25–1.00 g L^−1^) were added into the synthetic solution under agitation (200 rpm). After 30 min of agitation, the solution pH was adjusted to 5.5 ± 0.1. Then La(NO_3_)_3_·6H_2_O was added to target a La/F molar ratio of 1:3.00. After 1 h of reaction, the solution was vacuum filtrated. The filtration residue and filtrate were analyzed as mentioned above. To confirm the presence or absence of LaF_*x*_^3−*x*^ or LaF_3_·LaF_*x*_^3−*x*^, extra NaF (0.133 g) was added to the filtrate, and whether precipitate formed was checked.

## Results and Discussion

### Aqueous Equilibrium Diagram

**FIGURE 1:**
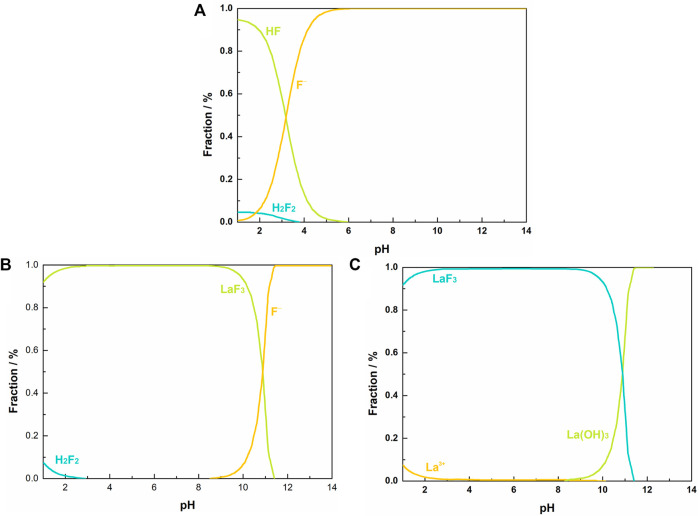
Aqueous equilibrium diagram: **(A)** fraction of fluorine species in F-H2O system at 298 K, [F]T = 0.01 M; fraction of fluorine species **(B)** and lanthanum species **(C)** in La-F-Cl-H2O system at 298 K, [La]T = 0.0053 M, [F]T = 0.0158 M, [Cl]T = 0.0338 M.

Figure 1A shows the distribution of fluorine species in F-H_2_O equilibrium system. At pH < 4.0, fluorine element exists in the form of HF, F^−^ and H_2_F_2_. The proportion of H_2_F_2_ decreases as pH increases from 1.0 to 4.0 and descends to zero at pH > 4.0. As a weak acid, most of fluorine exists as HF at low pH range (Li et al., 2013). Over the pH range 1.0–5.5, the proportion of HF descends rapidly as pH increases, while the proportion of F^−^ exhibits a reverse variation. At pH > 5.5, HF becomes negligible, and almost all the fluorine exists as free F^−^. Figures 1B, C show the distribution of F and La species in La-F-Cl-H_2_O equilibrium system. The molar ratio of La/F in the system is set as 1:3.00, and the total concentration of F and Cl is 0.0158 and 0.0338 M, respectively, same as the synthetic solution. As shown in Figures 1B,C, at pH < 3.0, a small part of fluorine exists as H_2_F_2_, while the majority of fluorine exists as LaF_3_. Over the pH range 3.0–8.3, almost all fluorine exists in the form of LaF_3_. Interestingly, as pH further increases (>8.3), La^3+^ tends to hydrolyze to La(OH)_3_, resulting in a decrease of LaF_3_ proportion. Based on the thermodynamic diagrams of La-F-Cl-H_2_O equilibrium system, it can be confirmed that at acidic environment (pH < 3.0), part of fluorine exists in the form of H_2_F_2_, which could hinder the combination of fluorine with La^3+^. Nevertheless, in the pH range over 8.3, La^3+^ hydrolysis reaction would compete with LaF_3_ precipitation reaction, resulting in a low fluoride removal and a high retaining fluorine concentration. Therefore, the pH range for fluoride removal using La^3+^ should be controlled between 5.5 and 8.3. Given the pH of the synthetic solution is about 2.0, in order to reduce the neutralization cost, the pH for fluoride removal is optimized to 5.5. Similarly, several literatures have also reported the largest adsorption capacity and highest defluorination efficiency of La-loaded adsorbents near pH of 5.5 (Cai et al., 2018).

### Fluoride Removal at La/F Molar Ratios ≥1:3.00

**FIGURE 2:**
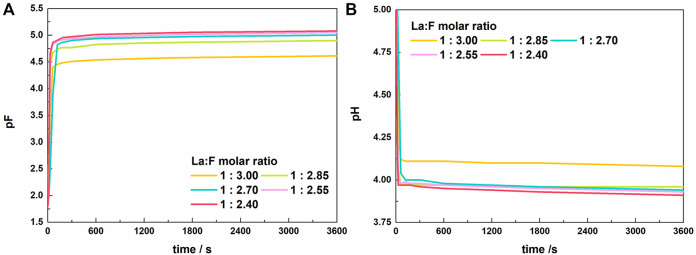
Variations of pF **(A)** and pH **(B)** of the solution with time after adding lanthanum nitrate to the fluorine-containing synthetic solutions with different La/F molar ratios (La/F ≥ 1:3.00).

Figure 2 shows the variation of solution pF and pH with time during the reaction at La/F molar ratios ≥1:3.00. At the initial stage, pF increased rapidly, indicating a sharp decreasing of free F^−^ concentration (Figure 2A). Meanwhile, the solution pH value exhibits a quick drop (Figure 2B). The decreasing pH could be explained by the enhanced ionization of HF due to the decreasing concentration of free F^−^, which results in a larger H^+^ activity. When the reaction time exceeds 300 s, the solution pF and pH change slightly, suggesting an equilibrium state obtained between La^3+^ and fluorine species. As the La/F molar ratio increases from 1:3.00 to 1:2.85, the stable pF increases apparently, and the pH exhibits a reverse change. This result indicates that addition of excessive La^3+^ can promote the combination of F^−^ and La. However, when the La/F molar ratio further increases, the stable pF increases slightly, indicating that increasing lanthanum dose has a small effect on the stable pF at La/F molar ratio ≥1:2.85.

As mentioned above, during the fluoride removal at La/F molar ratios ≥1:3.00, the stable pF is larger than 4.5, namely, the free F^−^ concentration could be reduced to below 0.5 mg L^−1^. However, after 1 h of reaction the solutions with La/F molar ratios ≥1:3.00 remained clear and transparent (shown in Supplementary Figure S1). During the vacuum filtration using inorganic membranes with a pore diameter of 0.22 μm, the mass of filtration residue collected from five solutions with different La/F molar ratios (≥1:3.00) is neglectable.

Due to the low free F^−^ concentration and the absence of precipitate, it is reasonable to assume that most of fluorine retains in the solution as soluble species. In order to verify this assumption, extra NaF (0.133 g) was added to each solution. The digital photos of solutions after adding extral NaF are shown in Supplementary Figure S1. Interestingly, flocculent precipitates formed immediately upon the addition of extra NaF. The XRD pattern and SEM image of the precipitates are shown in Supplementary Figure S2. It is apparent that characteristic peaks of LaF_3_ are observed in the XRD pattern of the precipitates (Supplementary Figure S2A), signifying the formation of LaF_3_ with extra addition of NaF. As shown in Supplementary Figure S2B, LaF_3_ precipitates exist as irregular crystalline bulks and amorphous particles simultaneously. The crystalline bulks may be resulted from the recrystallization of amorphous floccules during the vacuum filtration and drying process (Hermans and Weidinger., 1946). The mass of precipitates formed by adding extra NaF in different solutions is shown in Supplementary Figure S3. It is found that the mass of precipitates is close to the theoretical LaF_3_ production. These results confirm that at La/F molar ratio ≥1:3.00, fluorine retains in the solution as soluble species. However, reducing the La/F molar ratio by adding extra NaF could facilitate the formation of LaF_3_.

Considering the fact that fluoride ion could complex La^3+^, which results in the formation of LaF^2+^ and LaF_2_^+^, it could be inferred that in aqueous solutions with La/F molar ratios ≥1:3.00, most of fluorine retains in solution as LaF_*x*_^3−*x*^ complexes. Similarly, Antuñano et al. (2016) and Liu et al. (2020) reported the presence of AlF_*n*_^3−*n*^ complexes in Al-F-H_2_O system. Table 2 shows the distribution of fluorine in the solution after 1h of reaction with La/F molar ratios ≥1:3.00. It could be found that over 99% fluorine retains in solution as LaF_*x*_^3−*x*^. As the La/F molar ratio increases, the proportion of LaF_*x*_^3−*x*^ slightly increases. Since all fluorine retains in the solution in the form of LaF_*x*_^3−*x*^ or free F^−^, the fluoride removal efficiencies of solutions with La/F molar ratios ≥1:3.00 are all zero.

**TABLE 2:** Distribution of fluoride element in aqueous solutions with La/F molar ratio ≥1:3 after reaction for 1 h.

La/F molar ratio	Free F^−^			LaF_*x*_^3-*x*^		Retaining fluoride^c^	Removal
	*C* ^a^/mg L^−1^	Mass/mg	Proportion/%	Mass^b^/mg	Proportion/%	mg L^−1^	%
1:3.00	0.45	0.090	0.15	59.910	99.85	300	0
1:2.85	0.26	0.052	0.09	59.948	99.91	300	0
1:2.70	0.20	0.040	0.07	59.960	99.93	300	0
1:2.55	0.18	0.036	0.06	59.964	99.94	300	0
1:2.40	0.16	0.032	0.05	59.968	99.95	300	0

a Calculated with final pF of filtrate measured at pH 5.5 ± 0.1.

b Calculated with the fluorine balance $\left(60\;mg-mass_{free\;F-}-mass_{filtration\;residues}\times \frac{57}{196}\right)$ .

c Including free F^−^and LaF_*x*_^3-*x*^.

### Fluoride Removal at La/F Molar Ratios ≤1:3.00

It is confirmed that at La/F molar ratios ≥1:3.00 the fluorine could be not removed in the form of LaF_3_ precipitates. Therefore, it is necessary to investigate the chemical behavior of fluorine at La/F molar ratios ≤1:3.00.

**FIGURE 3:**
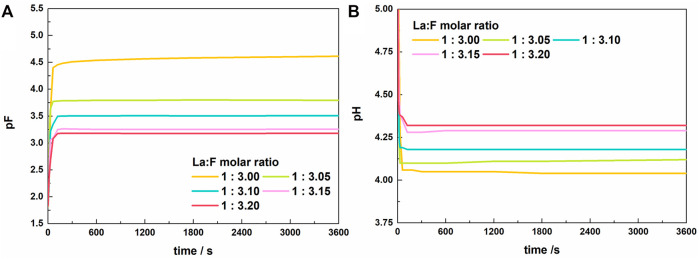
Variations of pF **(A)** and pH **(B)** of the solutions with time after adding lanthanum nitrate to the fluorine-containing synthetic solutions with different La/F molar ratios (La/F ≤ 1:3.00).

Figure 3 shows the variation of pF and pH with time in solutions with La/F molar ratios ≤1:3.00. Similar to the results obtained in solutions with La/F molar ratios ≥1:3.00, the reaction between La^3+^ and fluorine get an equilibrium state in 5 min. The stable pF decreases as the La/F molar ratio decreases, while the final pH shows the reverse change. Notably, at La/F molar ratio ≤1:3.00, the La/F molar ratio has a more significant influence on the stable pH and pF of the solutions. Specifically, as La/F molar ratio decreases from 1:3.00 to 1:3.05, the stable pF decreases from 4.50 to 3.75, indicating a much higher concentration of free F^−^ retaining in the solution.

Supplementary Figure S4 shows the photos of the solutions with different La/F molar ratios (≤1:3.00) after 1 h of reaction. It is apparent that a large number of white floccules suspend in the solutions with La/F molar ratios ≤1:3.05. As La/F molar ratio decreases, the solution becomes more and more turbid. It is worth noting that after standing for 2 h, the suspended solids could not settle. Therefore, it can be inferred that colloidal solutions were formed at La/F molar ratio ≤1:3.05. The formation of colloidal solution could be explained as follows. As mentioned above, in solutions at La/F ≥ 1:3.00, the insufficient fluorine results in formation of LaF_*x*_^3−*x*^, such as LaF^2+^ and LaF_2_^+^. In solution of La/F ≤ 1:3.05, the presence of sufficient fluorine promotes the formation of LaF_3_. However, the coexisting LaF_*x*_^3−*x*^ tends to adsorb on the surface of LaF_3_, resulting in negative charges on the surface of suspended solids (LaF_3_·LaF_*x*_^3−*x*^). This would enhance the stability of suspended solids and result in the formation of a colloidal solution.

**FIGURE 4:**
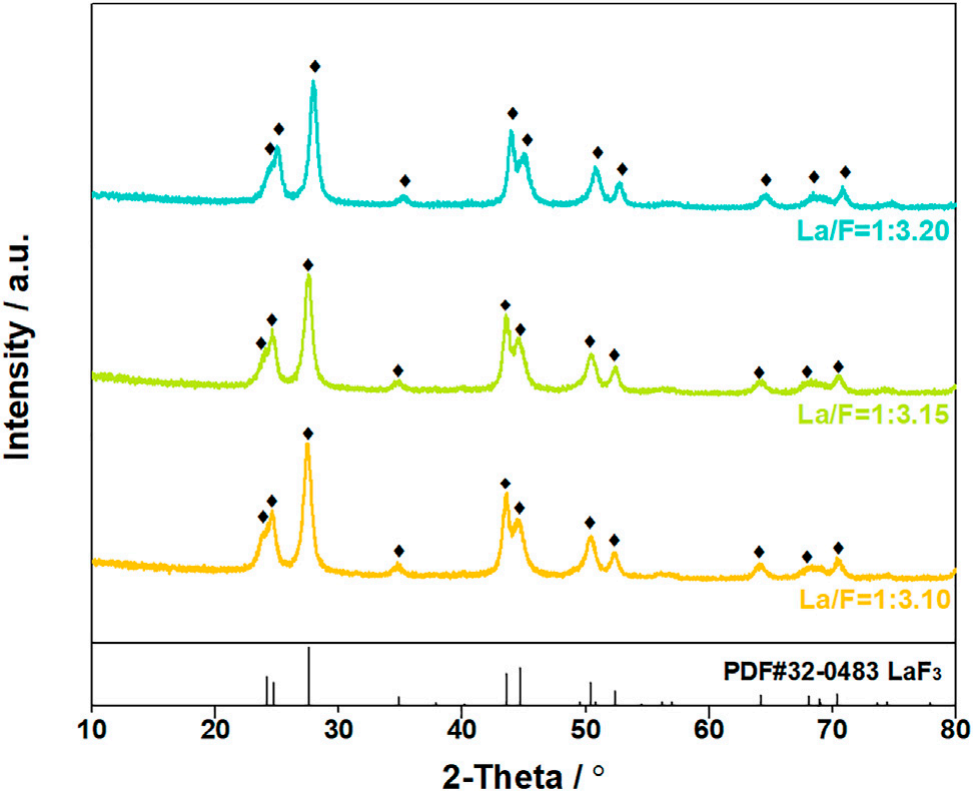
XRD patterns of filtration residues obtained in solutions at La/F molar ratio of 1:3.10, 1:3.15 and 1:3.20 after reaction for 1 h.

**FIGURE 5:**
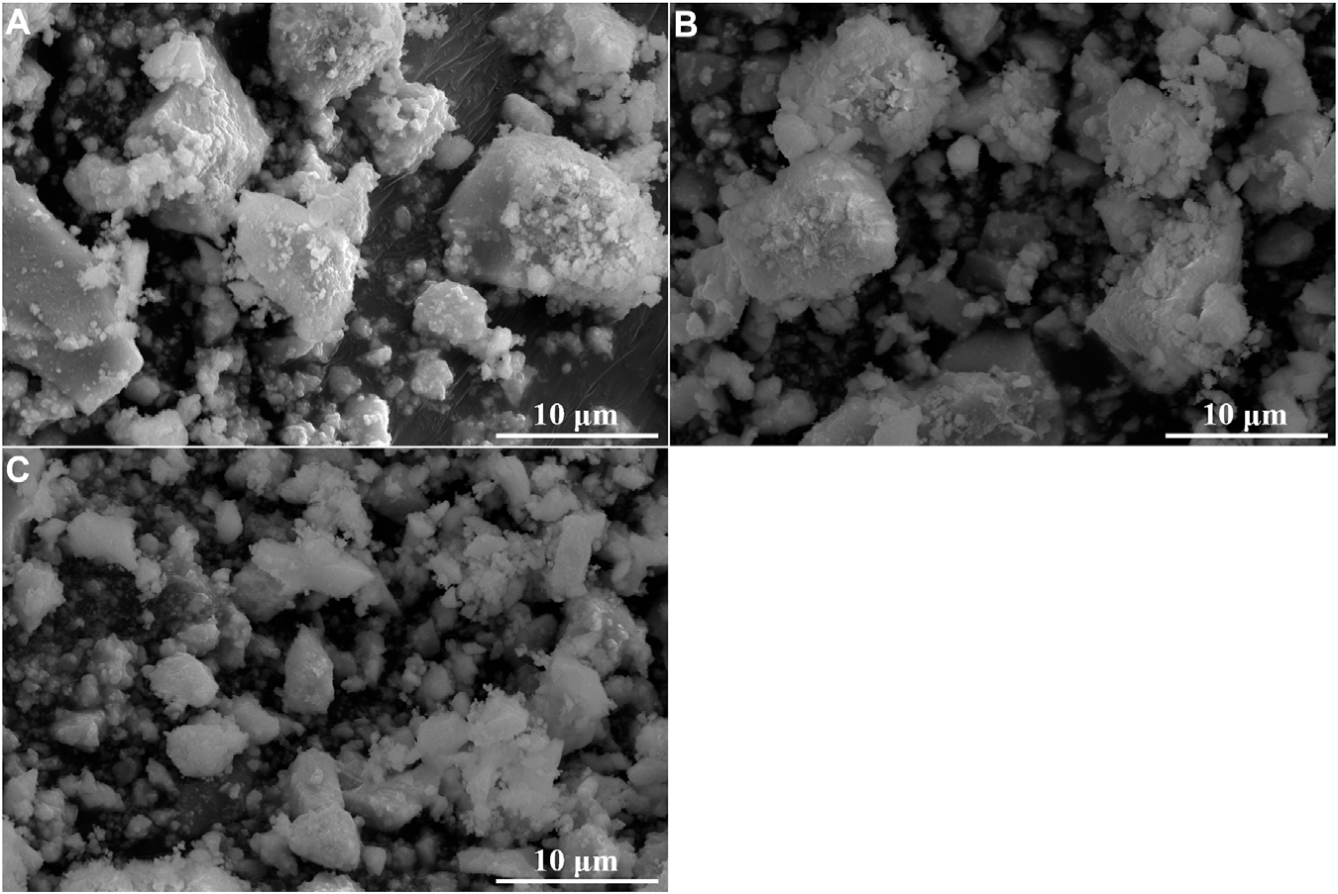
SEM images of filtration residues obtained in solutions with La/F molar ratios of 1:3.10 **(A)**, 1:3.15 **(B)** and 1:3.20 **(C)** after reaction for 1 h.

The suspended solids were collected by vacuum filtration using inorganic membrane. Interestingly, filtration residues could only be obtained from solutions with La/F molar ratio ≤1:3.10. The XRD patterns and SEM images of the filtration residues obtained from solutions with La/F molar ratio of 1:3.10, 1:3.15 and 1:3.20 are shown in Figures 4, 5, respectively. It can be found that the main phase of the filtration residues obtained in three solutions is LaF_3_. There is no other apparent characteristic peak, signifying the high purity of LaF_3_ precipitates. As shown in Figure 5, the LaF_3_ residues presents two kinds of structures, part of LaF_3_ exists as irregular bulks with high crystalline, while the others exhibit amorphous structures. As La/F molar ratio decreases, excessive fluoride accelerates the formation of LaF_3_, resulting in smaller particle size and a larger crystalline degree of LaF_3_. It is noteworthy that, at La/F molar ratio of 1:3.15 and 1:3.20, the filtrate obtained in vacuum filtration remains light pale, suggesting a small number of suspended solids remain in the solution. This could be explained with the smaller size of colloid particles formed with large fluorine excess coefficient, which could not be intercepted by the filtrate cake and membrane.

**FIGURE 6:**
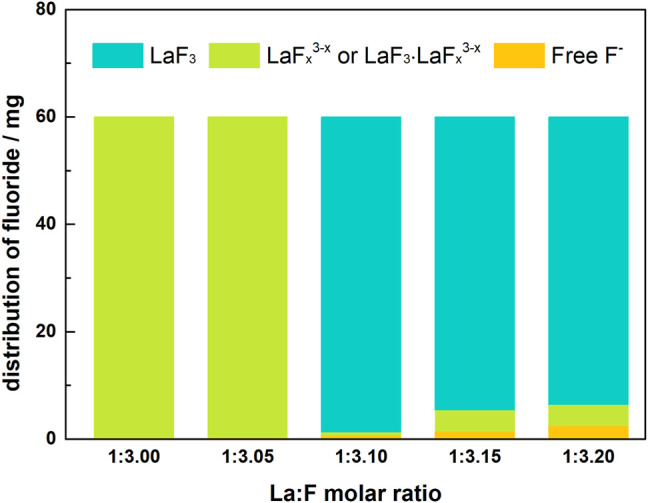
Distribution of fluorine element in solutions with different La/F molar ratios (≤1:3.00).

Table 3; Figure 6 show the distribution of fluorine in solutions with La/F molar ratios ≤1:3.00 after 1 h of reaction. It could be found that, as La/F molar ratio decreases (the excess coefficient of fluorine increases), the free F^−^ concentration of the equilibrium solution increases. At La/F molar ratio of 1:3.00 and 1:3.05, no fluorine is removed from the solution in the form of filtration residues, and over 98% fluorine retains in solution as LaF_*x*_^3−*x*^ or LaF_3_·LaF_*x*_^3−*x*^. Remarkably, at La/F molar ratio of 1:3.10, about 97.86% fluorine is removed from the aqueous system in the form of filtration residue, and the retaining fluoride concentration is about 6.42 mg L^−1^, slightly higher than the emission standard established in GB25467-2010. As La/F molar ratio further decreases, the proportion of free F^−^ increases obviously, while the proportion of filtration residues decreases, resulting in a higher retaining free F^−^ concentration and a decreasing fluoride removal efficiency. Specifically, when La/F molar ratio increases to 1:3.20, the retaining fluoride concentration is about 32.16 mg L^−1^, much higher than the emission standard. Although the presence of excess fluorine could facilitate the precipitation of LaF_3_, it leads to a significant increase in the retaining free F^−^ concentration and fine suspended colloid particles (LaF_3_·LaF_*x*_^3−*x*^). Therefore, the La/F molar ratio is optimized as 1:3.10 for precipitating fluoride using lanthanum salts.

**TABLE 3:** Distribution of fluorine element in aqueous solutions with La/F molar ratios ≤1:3.00 after reaction for 1 h.

La/F molar ratio	Free F^−^			LaF_*x*_^3−*x*^ or fine LaF_3_·LaF_*x*_^3-*x*^		Filtration residues			Retaining fluoride^d^	Removal
	*C* ^a^(mg L^−1^)	Mass (mg)	(%)	Mass^b^(mg)	(%)	Mass^c^ (mg)	Filtration time (min)	(%)	mg L^−1^	%
1:3.00	0.51	0.10	0.17	59.90	99.83	0	-	0	300	0
1:3.05	1.19	0.24	0.40	59.76	98.60	0	-	0	300	0
1:3.10	3.30	0.66	1.10	0.62	1.03	58.72	158	97.86	6.42	97.86
1:3.15	7.14	1.43	2.38	3.98	6.63	54.59	95	90.98	27.06	90.98
1:3.20	12.35	2.47	4.12	3.96	6.60	53.57	76	89.28	32.16	89.28

a calculated with final pF of filtrate measured at pH 5.5 ± 0.1.

b calculated with the fluorine balance $\left(60\;mg-mass_{free\;F-}-mass_{filtration\;residues}\times \frac{57}{196}\right)$ .

c calculated with the mass of precipitate $\left(mass_{filtration\;residues}\times \frac{57}{196}\right)$ based on the assumption that precipitate contains LaF_3_ only.

d including free F^−^, LaF_*x*_^3−*x*^ and fine LaF_3_·LaF_*x*_^3-*x*^.

### Chemical Reactions Between F^−^ and La^3+^

**FIGURE 7:**
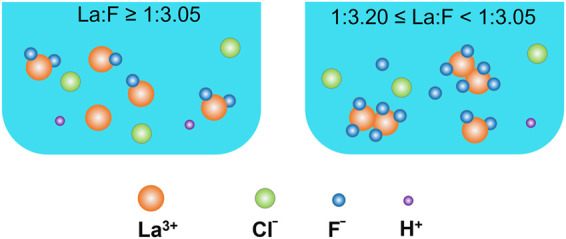
Schematic diagrams of species in La-F-Cl-H_2_O aqueous system.

Based on the above results, it can be found that the chemical reactions between F^−^ and La^3+^ are sensitive to the La/F molar ratio. As shown in Figure 7, at La/F molar ratio ≥1:3.05, the fluorine is relatively insufficient, there is not enough fluorine participate in the precipitation reaction (Eq. 16), resulting in the formation of LaF_2_^+^ (Eq. 17), or even LaF^2+^ (Eq. 18). Under this condition, although the free F^−^ concentration in the aqueous system can be reduced to a very low level, most of fluorine retains in the solution in the form of LaF_*x*_^3−*x*^ complexes, resulting in the failure of fluorine removal. At 1:3.20 ≤ La:F < 1:3.05, sufficient fluoride enhances the precipitation of LaF_3_. However, coexisting LaF_*x*_^3−*x*^ in the aqueous solution could adsorb on the surface of LaF_3_, resulting in the formation of colloidal solution with LaF_3_·LaF_*x*_^3−*x*^ suspended solids (Eqs 19–20).

$${\text{La}}^{3+}+{3\text{F}}^{-}={\text{LaF}}_{3}$$ (16)

$${\text{La}}^{3+}+2{\text{F}}^{-}={\text{LaF}}_{2}^{+}$$ (17)

$${\text{La}}^{3+}+{\text{F}}^{-}={\text{LaF}}^{2+}$$ (18)

$${\text{LaF}}_{3}+{\text{LaF}}_{2}^{+}={\text{LaF}}_{3}\cdot {\text{LaF}}_{2}^{+}$$ (19)

$${\text{LaF}}_{3}+{\text{LaF}}^{2+}={\text{LaF}}_{3}\cdot {\text{LaF}}^{2+}$$ (20)

### Coagulation Removal of Fluoride Using La^3+^ and Na_2_SiO_3_

At the optimized La/F molar ratio of 1:3.10, the retaining fluorine concentration in the filtrate is 6.42 mg L^−1^, exceeding the emission standard level (GB25467-2010). Therefore, chemical precipitation using lanthanum salt fails to sufficiently remove fluoride from aqueous solution. Given that fluorine could exists in the form of positively charged LaF_*x*_^3−*x*^ or LaF_3_·LaF_*x*_^3−*x*^ (*x* = 1 or 2), SiO_2_·*n*H_2_O, as a well-known negatively charged colloidal particle (Lin et al., 2018), is introduced to facilitate the coagulation of LaF_*x*_^3−*x*^ and LaF_3_·LaF_*x*_^3−*x*^ based on electrostatic interaction. In practice, Na_2_SiO_3_ was firstly added into the synthetic solution. After 0.5 h of agitation, the solution pH was adjusted to 5.5 ± 0.1 to produce active colloidal particles (SiO_2_·*n*H_2_O) *in situ*. Afterwards, the lanthanum salt is added to target a La/F molar ratio of 1:3.00, rather than 1:3.10 in order to reducing the retaining free F^−^ concentration.

Supplementary Figure S5 shows the digital photos of solutions with different doses of Na_2_SiO_3_ after 1 h of reaction. In the absence of Na_2_SiO_3_ (Supplementary Figure S5A), the solution remains clear and transparent. Correspondingly, during the vacuum filtration, there is no filtration residues obtained from this solution. In the presence of 0.25 g L^−1^ Na_2_SiO_3_ (Supplementary Figure S5B), about 0.10 g L^−1^ filtration residues were collected from the solution. As Na_2_SiO_3_ dose further increases, the solutions at equilibrium state become more and more turbid, signifying the formation of suspended particles. Consequently, much more filtration residues were obtained from these solutions.

**FIGURE 8:**
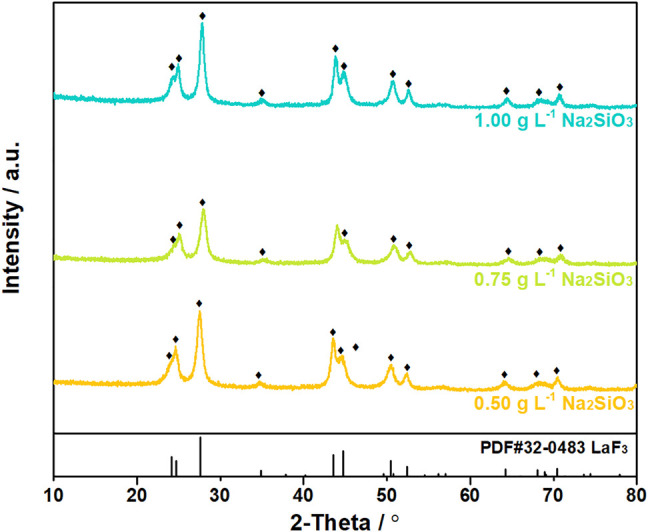
XRD patterns of filtration residues obtained from solutions with La/F molar ratio of 1:3.00 after 1 h reaction in the presence of different doses of Na_2_SiO_3_.

**FIGURE 9:**
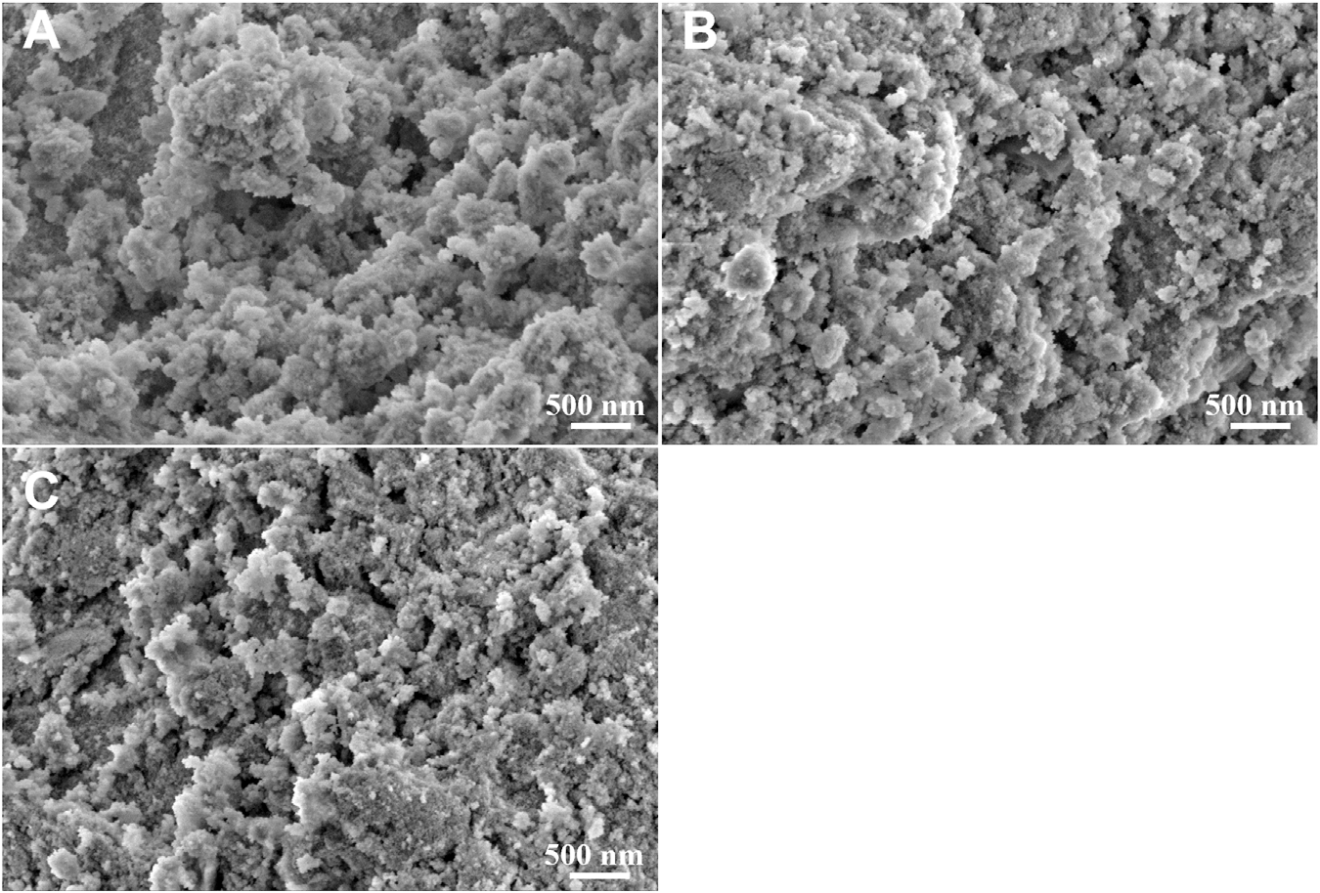
SEM images of filtration residues obtained at La/F molar ratios of 1:3.00 with different doses of Na_2_SiO_3_. **(A)** 0.50 g L^−1^, **(B)** 0.75 g L^−1^, (**C**) 1.00 g L^−1^.

The XRD patterns and SEM images of the filtration residues were shown in Figures 8, 9. In the XRD patterns of filtration residues obtained from solutions with 0.50–1.00 g L^−1^ Na_2_SiO_3_, only the featured peaks of LaF_3_ appears, demonstrating the main phase of the filtration residues is LaF_3_. The absence of characteristic peaks of SiO_2_ may be explained by the low content of SiO_2_·*n*H_2_O or the amorphous structure of SiO_2_·*n*H_2_O. The morphologies of filtration residues obtained with Na_2_SiO_3_ addition are shown in Figure 9. It can be found that the presence of Na_2_SiO_3_ has an obvious effect on the structures of filtrate residues. A large number of fine irregular particles agglomerates, resulting in the lumps in large size (≥5 μm). However, the dose of Na_2_SiO_3_ does not show obvious influence on the morphologies of filtration residues.

**FIGURE 10:**
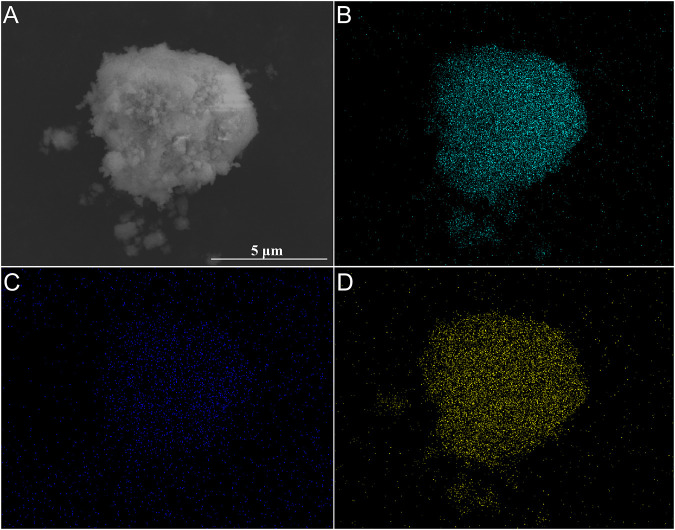
SEM image **(A)** and elements mapping **(B–D)** of filtration residues obtained at La/F molar ratios of 1:3.00 in the presence of 0.50 g L^−1^ Na_2_SiO_3_. **(B)** F, **(C)** Si, **(D)** La.

Figure 10 shows the distribution of F, Si and La element on the surface of filtration residues obtained from solution with La/F molar ratio of 1:3.00 and 0.50 g L^−1^ Na_2_SiO_3_. It is observed that the F and La element is uniformly distributed on the surface of filtration residues, further demonstrating the formation of LaF_3_ precipitates. In spite of the absence of featured peaks of SiO_2_ in the XRD pattern, the Si element is detected on the surface of filtration residues, which testifies the participation of colloidal particles (SiO_2_·*n*H_2_O) in the coagulation of LaF_3_. The EDS results indicate that the contents of Si in filtration residues are 2.87 wt%, 3.67 wt% and 4.25 wt%, corresponding to the addition of 0.50 g L^−1^, 0.75 g L^−1^, and 1.00 g L^−1^ Na_2_SiO_3_, respectively.

**FIGURE 11:**
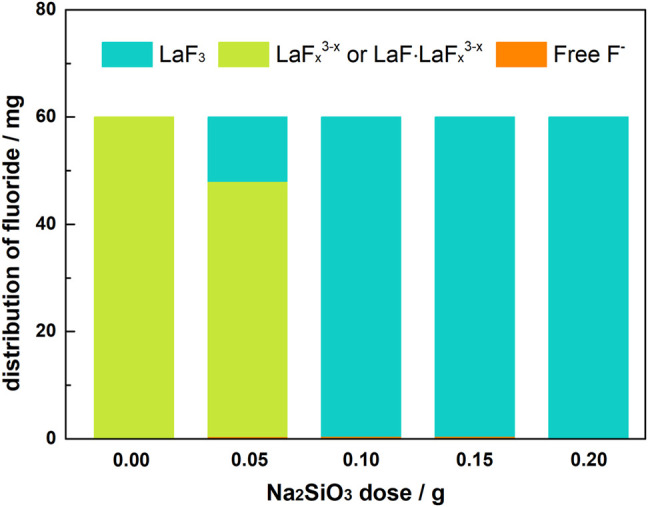
Distribution of fluorine element in aqueous solutions (La/F molar ratio = 1:3.00) after 1 h reaction in the presence of different doses of Na_2_SiO_3_.

Table 4; Figure 11 shows the distribution of fluorine element in solutions with La/F molar ratio of 1:3.00 and different doses of Na_2_SiO_3_. In the absence of Na_2_SiO_3_, even though free F^−^ concentration could be reduced to 0.51 mg L^−1^, nearly 99.83% fluorine retains in the solution as LaF_*x*_^3−*x*^. When Na_2_SiO_3_ dose reaches 0.25 g L^−1^, about 20.12% fluorine is removed from the solution in the form of filtration residues, and 79.25% remains in the solution in the form of LaF_*x*_^3−*x*^. As Na_2_SiO_3_ dose increases to 0.50 g L^−1^, a fluorine removal efficiency of 99.25% could be obtained. When adding extra NaF into the filtrate, the solution keeps clear and transparent, proving the absence of LaF_*x*_^3−*x*^ or LaF_3_·LaF_*x*_^3−*x*^. The retaining fluoride concentration in equilibrium solution is 2.24 mg L^−1^, lower than the emission standard established in GB25467-2010. As Na_2_SiO_3_ dose further increases, the fluorine removal efficiency ascends slightly, and the retaining fluoride concentration could be reduced to 0.8 mg L^−1^, lower than the limited fluorine level established by the WHO. However, it is noteworthy that, at a Na_2_SiO_3_ dose ≥0.50 g L^−1^, the filtration time is longer than 5 h, which could be explained by the formation of a large amount of sticky SiO_2_·*n*H_2_O colloidal particles.

**TABLE 4:** Distribution of fluorine element in aqueous solutions (La/F molar ratio = 1:3.00) after 1 h of reaction in the presence of Na_2_SiO_3_.

Na_2_SiO_3_ dose (g L^−1^)	Free F^−^			LaF_*x*_^3−*x*^ or fine LaF_3_·LaF_*x*_^3-*x*^		Filtration residues			Retaining fluoride^d^	Removal
	*C* ^a^ (mg L^−1^)	Mass (mg)	(%)	Mass (mg)	(%)	Mass (mg)	Filtration time (min)	(%)	(mg L^−1^)	%
0	0.51	0.10	0.17	59.90^b^	99.83	0	-	0	300.00	0
0.25	1.89	0.38	0.63	47.55^b^	79.25	12.07	2.3	20.12	271.15	20.12
0.50	2.24	0.45	0.75	0	0	59.55^c^	340	99.25	2.24	99.25
0.75	2.33	0.47	0.78	0	0	59.53^c^	362	99.22	2.33	99.22
1.00	0.80	0.16	0.27	0	0	59.84^c^	325	99.73	0.80	99.73

a calculated with final pF of filtrate measured at pH 5.5 ± 0.1.

b Calculated with the fluorine balance $\left(60\;mg-mass_{free\;F-}-mass_{filtration\;residues}\times \frac{57}{196}\right)$ based on the assumption that precipitate contains LaF_3_ only (Si is neglected).

c Calculated with the fluorine balance $\left(60\;mg-mass_{free\;F-}\right)$ , the absence of LaF_*x*_^3−*x*^ or LaF_3_·LaF_*x*_^3−*x*^ is proved by adding extra NaF.

d Including free F^−^,LaF_*x*_^3−*x*^ and fine LaF_3_·LaF_*x*_^3-*x*^.

Above results verify our assumption that the presence of negatively charged SiO_2_·*n*H_2_O colloidal particles could interact with positively charged LaF_*x*_^3−*x*^ and LaF_3_·LaF_*x*_^3−*x*^, promoting their aggregation and settlement. It is demonstrated that coagulation removal of fluoride could be achieved through adding Na_2_SiO_3_ and lanthanum salts. However, the presence of SiO_2_·*n*H_2_O colloidal particles render the difficulty in solid/liquid separation, the operation parameters for coagulation removal of fluoride using Na_2_SiO_3_ and lanthanum salts should be optimized in future works.

## Conclusion

Pyrometallurgical treatment of low-grade sphalerite ores and zinc-bearing dusts discharges a large amount of flue gas containing HF and HCl. During the flue gas scrubbing step, fluorine and chlorine transfer into the liquid phase, yielding a large amount of fluorine-containing scrubbing wastewater. In this work, precipitation removal and coagulation removal of fluoride from flue gas scrubbing wastewater (300 mg L^−1^ fluorine) using lanthanum salt is investigated.

The chemical reaction between La^3+^ and F^−^ has been discussed based on the distribution of fluorine in solutions with different La/F molar ratios. At acidic environment (pH ≤ 4.0), part of fluorine exists as HF and H_2_F_2_, retarding the combination of La^3+^ and fluorine. Nonetheless, at pH > 8.3, La^3+^ hydrolysis reaction would compete with LaF_3_ precipitation reaction, resulting in a low fluoride removal. Consequently, the pH for fluoride removal is optimized to 5.5. At La/F molar ratio ≥1:3.05, most of fluorine retains in the solution in the form of LaF_*x*_^3−*x*^ complexes, resulting in the failure of fluorine removal. At 1:3.20 ≤ La:F ratio <1:3.05, sufficient fluoride enhances the precipitation of LaF_3_. However, LaF_*x*_^3−*x*^ coexisting in the aqueous solution could adsorb on the surface of LaF_3_, resulting in the formation of colloidal solution with large numbers of LaF_3_·LaF_*x*_^3−*x*^ suspended solids. In summary, at optimized La/F molar ratio of 1:3.10, about 97.86% fluorine is removed from the aqueous system in the form of filtration residue, and the retaining fluoride concentration is about 6.42 mg L^−1^, slightly higher than the emission standard established in GB25467-2010.

Considering the existing of positively charged LaF_*x*_^3−*x*^ and LaF_3_·LaF_*x*_^3−*x*^, coagulation removal of fluoride is proposed and investigated using lanthanum salts and negatively charged SiO_2_·nH_2_O colloidal particles (*in-situ* produced *via* Na_2_SiO_3_ hydrolysis at pH near 5.5). At a La/F molar ratio of 1:3.00 and Na_2_SiO_3_ dose of 0.50 g L^−1^, a fluoride removal of 99.25% is obtained with retaining fluorine concentration of 2.24 mg L^−1^. When Na_2_SiO_3_ dose increases to 0.20 g, the retaining fluorine concentration could be further reduced to 0.80 mg L^−1^, lower than the limited fluoride level established by the WHO. During the coagulation removal of fluoride using lanthanum salts and Na_2_SiO_3_, the presence of SiO_2_·*n*H_2_O colloidal particles render the difficulty in solid/liquid separation, the operation parameters for coagulation removal of fluoride should be optimized in future works.

Generally, coagulation removal of fluoride has a huge potential to be adopted in metallurgical industry due to high removal efficiency, low consumption of lanthanum salts, and relative low cost of lanthanum salts and Na_2_SiO_3_. However, the influence of impurities, especially anions such as SO_4_^2-^, Cl^−^, CO_3_^2-^, and NO_3_^−^, on the chemical behavior and distribution of fluoride during coagulation removal of fluoride should be fully evaluated before industrialized application of this process.

## Data Availability

The original contributions presented in the study are included in the article/Supplementary Material, further inquiries can be directed to the corresponding authors.
