# Supplementary material for: Efficient Coagulation Removal of Fluoride Using Lanthanum Salts: Distribution and Chemical Behavior of Fluorine
Source: Front Chem. 2022 Mar 4;10:859969. doi: 10.3389/fchem.2022.859969 (PMC8931680; doi:10.3389/fchem.2022.859969)
Supplement: Supplementary file 1 [file DataSheet1.docx]

*Supplemental Images*

Efficient Coagulation Removal of Fluoride Using Lanthanum Salts: Distribution and Chemical Behavior of Fluorine

*Xiaocong Zhong^1,2^, Chen Chen^1^, Kang Yan^1^, Shuiping Zhong^1^, Ruixiang Wang^1*^, Zhifeng Xu^3*^*

*^1^Faculty of Materials Metallurgy and Chemistry, Jiangxi University of Science and Technology, Ganzhou 341000, Jiangxi, China. ^2^State Key Laboratory of Separation and Comprehensive Utilization of Rare Metals, Guangzhou 510650, China. ^3^Jiangxi College of Applied Technology, Ganzhou 341000, Jiangxi, China.*

**** Correspondence:***

*Ruixiang Wang, E-mail: jxustpaper@163.com*

*Zhifeng Xu, E-mail: jxustxzf@163.com*


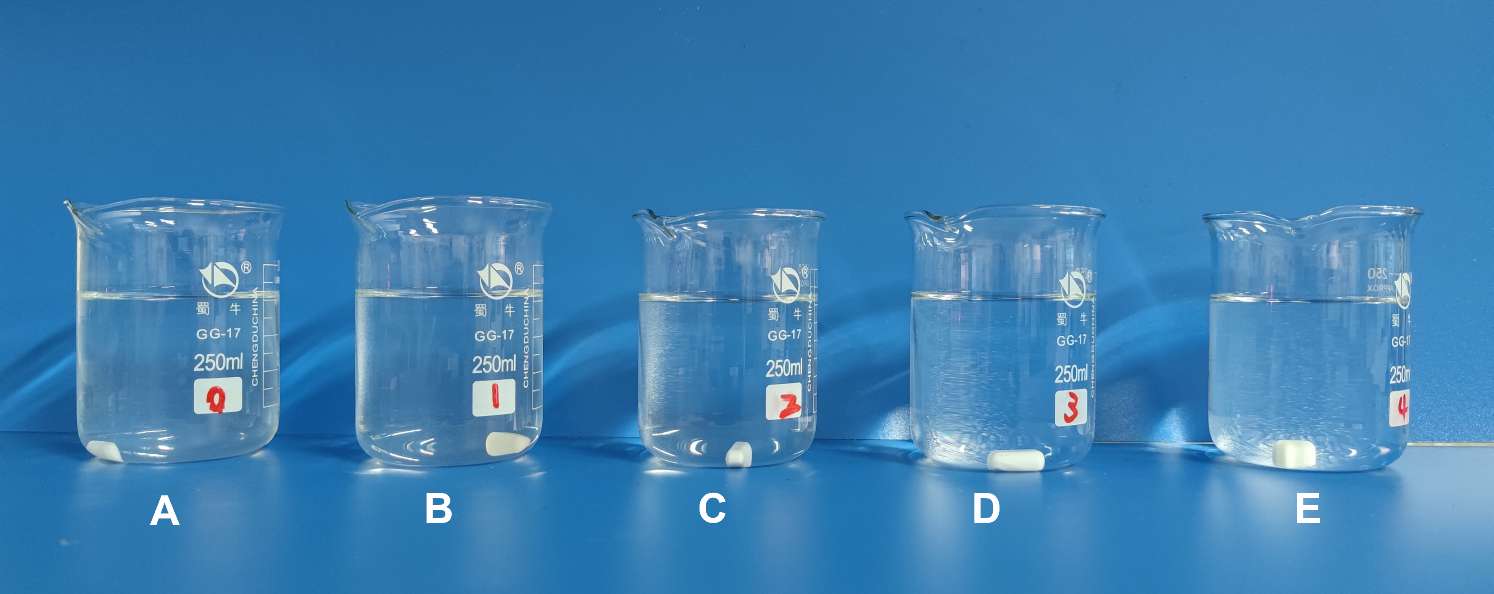


**FIGURE S1│** Photos of solutions after 1h of reaction with different La/F molar ratios. (**A**) La/F=1:3.00, (**B**) La/F=1:2.85, (**C**) La/F=1:2.70, (**D**) La/F=1:2.55, (**E**) La/F=1:2.40

***
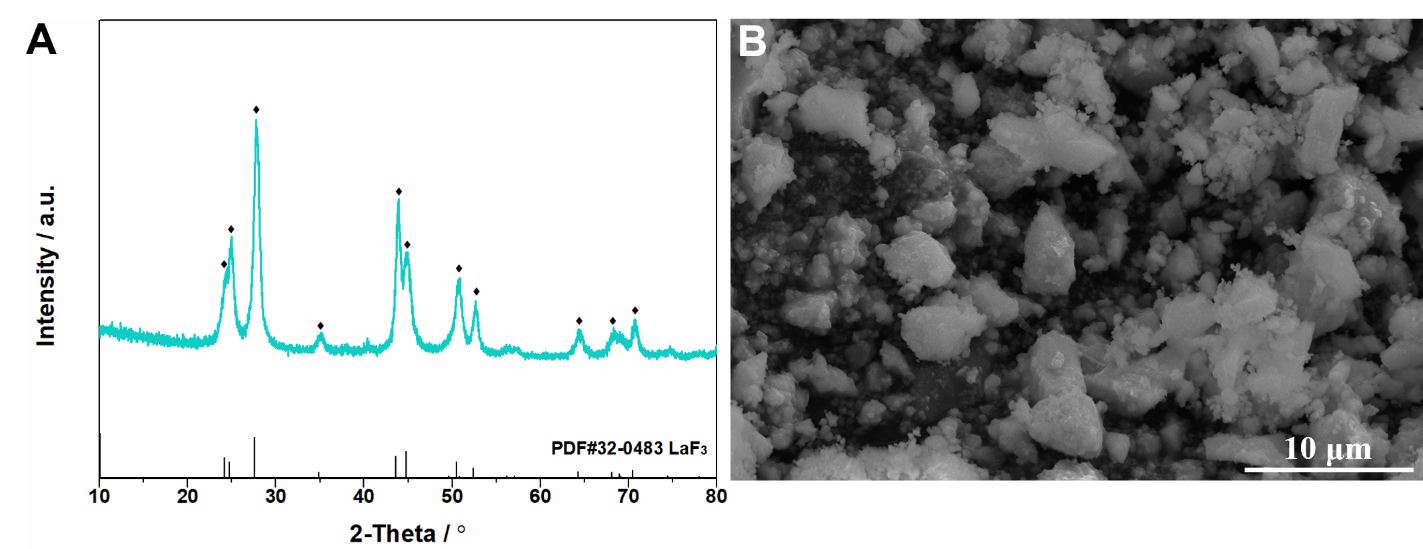
***

**FIGURE S2│** XRD pattern **(A)** and SEM image **(B)** of precipitates produced by adding extra NaF (0.133 g) into solution with La/F molar ratio of 1:3.00

***

***

**FIGURE S3│** The mass of precipitates formed by adding extra NaF (0.133 g) into solutions with La/F molar ratios ≥ 1:3.00


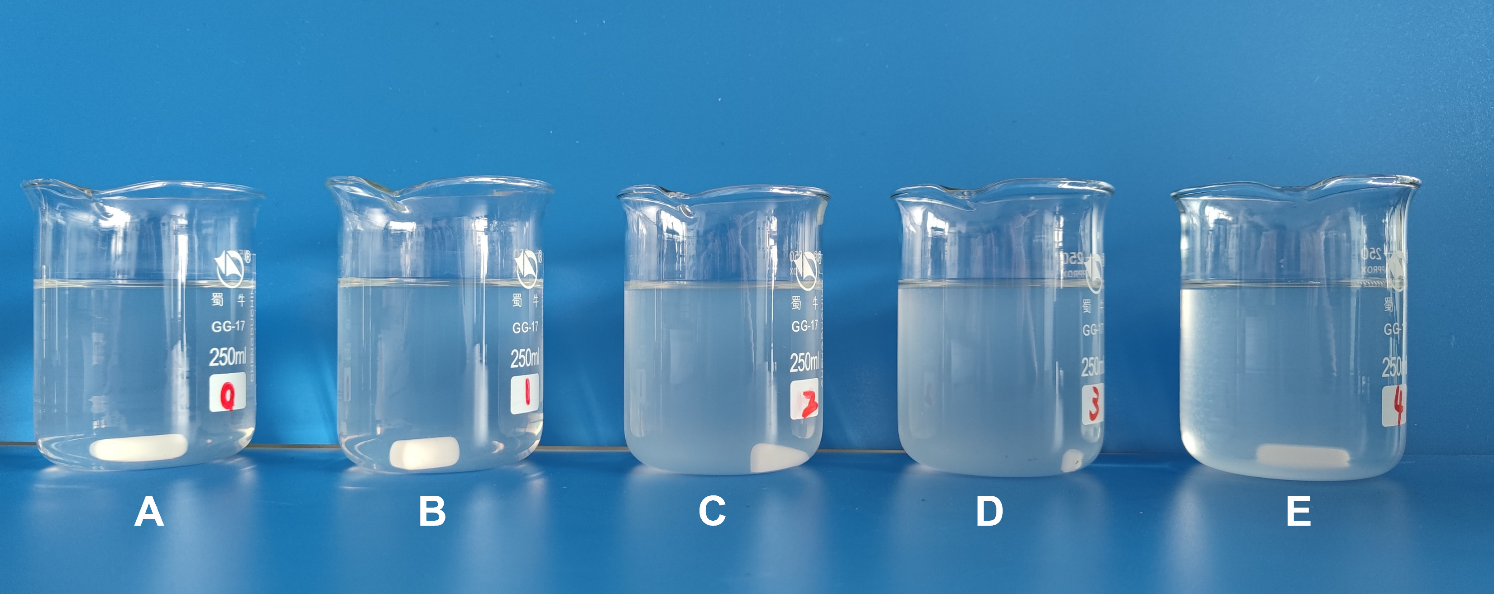


**FIGURE S4│**Photos of the solutions with different La/F ratios (≤ 1:3.00) after1 hour of reaction.


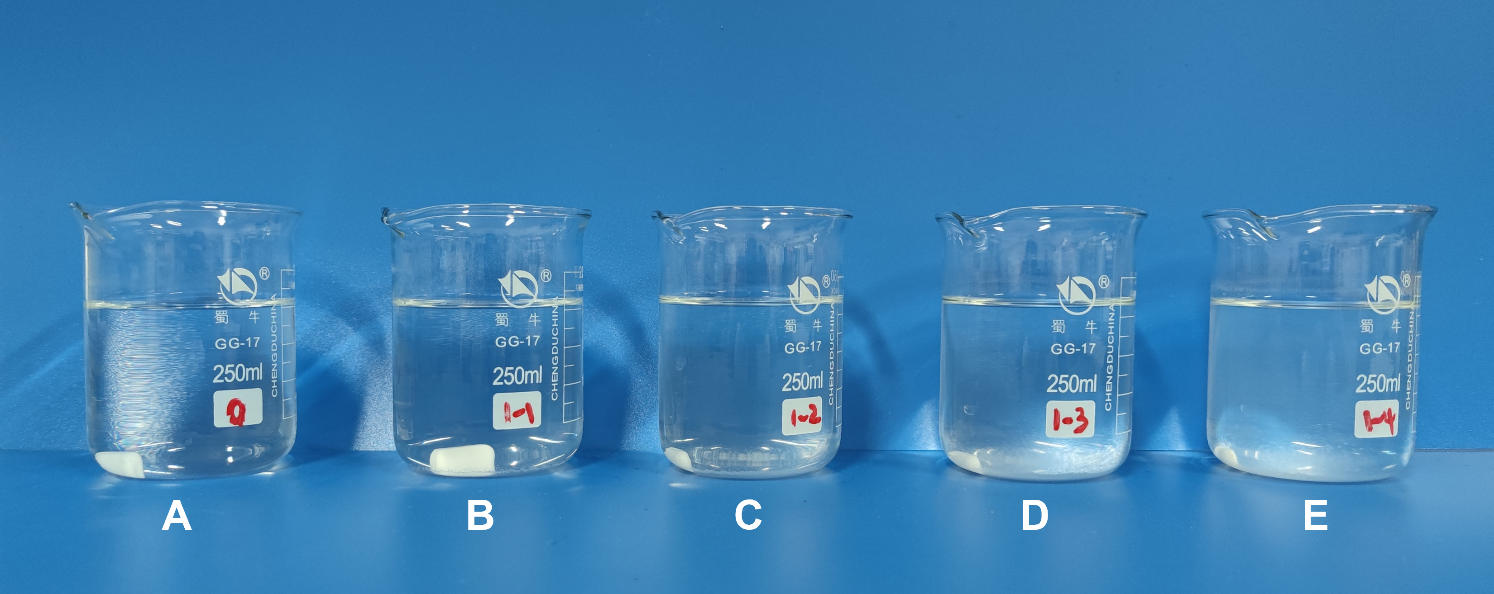


**FIGURE S5│** Photos of solutions with La/F ratio = 1:3.00 after 1h reaction in the presence of different doses of Na_2_SiO_3_. (**A**) 0, (**B**) 0.25 g L^-1^, (**C**) 0.50g L^-1^, (**D**) 0.75 g L^-1^, (**E**) 1.00 g L^-1^.
